# Racial and Ethnic Disparities in the Diagnosis and Care of Peripheral Artery Disease in the United States: A Systematic Review

**DOI:** 10.7759/cureus.104538

**Published:** 2026-03-02

**Authors:** Angela Ojo, Miriam A Okorie, Collins C Okeke, Desmond E Orie, Afamefuna O Onyeogulu, Emmanuel I Akinteye, Miliete T Berhe, Otutochukwu O Ike-Obioha, Omosimisola O Alli, Chinecherem C Ezema, Sylvahelen Okorienta, Mmesomachukwu O Amuluche, Euodia A Ugo-Ihanetu

**Affiliations:** 1 Internal Medicine, Afe Babalola University, Ado Ekiti, NGA; 2 Medicine and Surgery, Ebonyi State University, Abakaliki, NGA; 3 Internal Medicine, University of Port Harcourt Teaching Hospital, Port Harcourt, NGA; 4 Internal Medicine, Delta State University Teaching Hospital, Oghara, NGA; 5 Internal Medicine, Nnamdi Azikiwe University Teaching Hospital, Nnewi, NGA; 6 Medicine and Surgery, Abuad Multisystem Hospital, Ekiti, NGA; 7 Medicine, Ayder Comprehensive Specialized Hospital, Mekelle University, Mekelle, ETH; 8 Medicine and Surgery, University of Port Harcourt Teaching Hospital, Port Harcourt, NGA; 9 General Practice, Lagos State University College of Medicine, Lagos, NGA; 10 Integrative Medicine, Nnamdi Azikiwe University Teaching Hospital, Nnewi, NGA; 11 Public Health, Liberty University, Lynchburg, USA; 12 Internal Medicine, Rivers State University Teaching Hospital, Port Harcourt, NGA

**Keywords:** ethnic disparities, management, peripheral artery diseases, racial disparity, usa: united states of america

## Abstract

Peripheral artery disease (PAD) commonly affects the lower extremity and occurs due to the narrowing or blockage of vessels carrying blood from the heart to the legs. This review aims to examine racial and ethnic disparities in the diagnosis and management of PAD among adults in the United States by summarizing differences in diagnostic evaluation, treatment utilization, and management outcomes.

A search was done from inception to the 18th of November 2025 across PubMed and Google Scholar, and 1,372 articles were generated. Eight articles were included for the final analysis after applying the pre-defined eligibility criteria. We included an original article published in a peer-reviewed journal that discusses racial/ethnic disparities in the diagnosis and/management of peripheral artery disease among adults of different races in the United States of America. Joanna Briggs Institute (JBI) risk of bias critical appraisal tool for cohort studies.

Across the included studies, a total of 5,512,632 participants were reported, comprising 4,784,099 (86.78%) White patients, 576,711 (10.46%) Black patients, 51,408 (0.93%) Hispanic patients, and 100,414 (1.82%) patients from other racial and ethnic groups across the United States. Only one article discussed diagnostic testing among different racial groups and found that Black patients were more likely to undergo diagnostic evaluation for PAD. The majority of White patients underwent revascularization, revascularization combined with medical therapy, vascular bypass surgery, and medical therapy, whereas Black patients more frequently underwent amputation as the treatment for PAD.

In this review, Black patients consistently experienced higher rates of lower-limb amputation and lower utilization of revascularization and evidence-based medical therapies compared with White patients. Furthermore, limited data on diagnostic practices and the underrepresentation of Hispanic patients and patients from other racial and ethnic groups highlight an important research gap.

## Introduction and background

Peripheral artery disease (PAD) is a common manifestation of systemic atherosclerosis in the lower extremity, leading to narrowing or blockage of vessels carrying blood from the heart to the legs [1-3]. PAD occurs due to atherosclerosis, a progressive accumulation of lipids and inflammatory cells within the arterial wall. Atherosclerotic plaque builds up over time on the inside of arteries, leading to dilation of the arteries in the early stage of PAD to allow blood flow, the arteries cannot dilate any further, causing narrowing of the artery lumen by the atherosclerotic plaque [2]. The prevalence of PAD varies across sex, race, and ethnicity; however, the overall prevalence of PAD in the United States is estimated to be seven to 12 million, while 200 million globally [4,5]. Risk factors for PAD include smoking, high blood pressure, atherosclerosis, diabetes, age >60, and high cholesterol. PAD can manifest in different ways, including smooth, shiny skin, hair loss, muscle atrophy, cool skin to the touch, decreased or absent pulses in the feet, an ulcer on the leg or feet that does not heal, and cold or numb toes [1]. Despite the advances in medicine and technology, the outcomes from PAD, which include limb ischemia, limb amputation, and mortality, remain increased among a specific racial and ethnic group in the United States. The consequences of this disparity are higher among African Americans [6,7].

This review aims to examine racial and ethnic disparities in the diagnosis and management of peripheral artery disease (PAD) among adults in the United States by summarizing differences in diagnostic evaluation, treatment utilization, and management outcomes.

## Review

Methods

This systematic review was conducted in accordance with Preferred Reporting Items for systematic reviews and meta-analysis (PRISMA 2020). The study protocol was registered with PROSPERO (CRD420261285178). This study protocol was registered before data extraction.

Eligibility

Original article published in a peer-reviewed journal and discusses racial/ethnic disparities in the diagnosis and management of peripheral artery disease among adults of different races in the United States of America.

Exclusion Criteria

We excluded those that did not discuss racial/ethnic disparity or diagnosis and or management of peripheral artery disease in the United States. We also excluded patient <18 years of age, non-English articles, abstracts, reports, comments, surveys, case reports, case series, editorials, systematic reviews, and meta-analyses.

Search Strategy

A search was done from inception to the 18th of November 2025, on PubMed and Google Scholar(Displayed only 50 pages) databases with the following search phrases across the database; (Ethnic) OR (Racial) OR (Disparity) AND (Treatment) AND (Peripheral artery disease) AND (United States), “Ethnic" "Racial" "Disparity" "Treatment" "Peripheral artery disease" "United States". More details are shown in Appendix 1.

The search results from various databases were imported into Rayyan referencing manager [8], where duplicate, title, and abstract screening were carried out by three independent co-authors. Following abstract screening, the eligible articles were subjected to full-text screening using the pre-defined eligibility criteria. Disagreements were discussed among the authors, and another co-author will be invited if no resolution is reached. 

Data extraction from the eligible articles was done by four co-authors independently into a Google spreadsheet. The extracted baseline variables include: author's name, country, study year, sample size by race/ethnic, gender, mean age, and disparity in diagnosis and treatment.

Race and ethnicity were categorized according to the definitions used in the original studies. In this review, race was described as a socially shared physical trait (e.g., White or Black), whereas ethnicity was defined as shared cultural identity or heritage (e.g., Hispanic).

The primary outcome of this review is to evaluate the disparity in access to various treatment modalities among individuals of different races/ethnicities diagnosed with PAD in the United States. Our secondary outcome is evaluate the disparity in diagnostic testing and the rate of utilization of the treatment modalities of PAD.

Eligible articles underwent quality assessment using the Joanna Briggs Institute (JBI) risk of bias critical appraisal tool for cohort studies [9], as shown in Appendix 2. The included studies demonstrated strong methodological quality; all studies reported comparable groups, exposure, and outcome measurements, identification and adjustment for confounders, with appropriate statistical analyses. Some limitations were observed, as some of the included articles did not report the duration and completeness of their follow-up. None of the included articles mentioned ways to address incomplete follow-up. Also, the JBI critical appraisal tool does not have a standardized grading to report articles with high or low risk of bias.

Results

**Figure 1:**
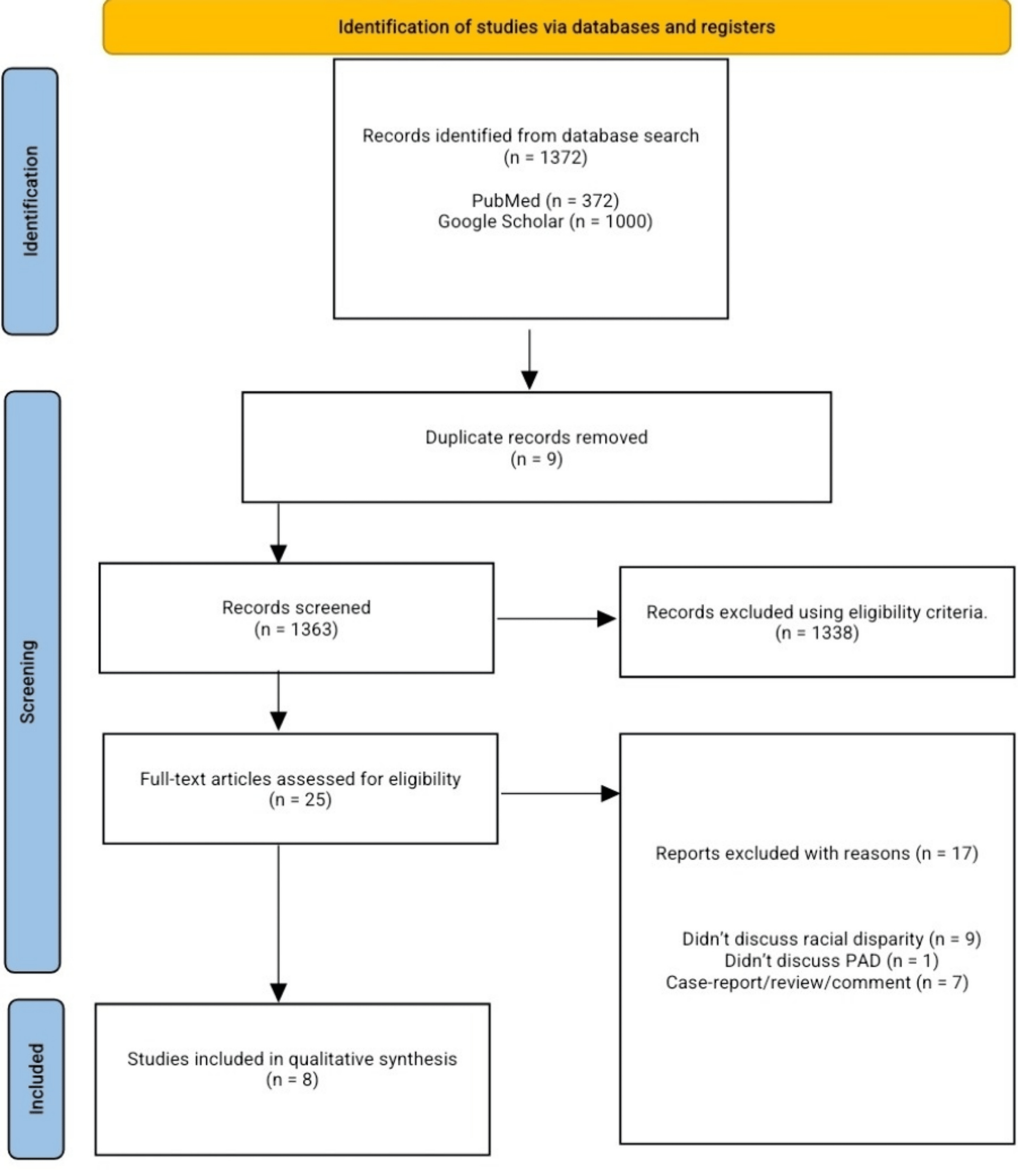
PRISMA flow diagram. PRISMA: Preferred Reporting Items for Systematic Reviews and Meta-Analysis, PAD: peripheral artery disease.

Our search across databases yielded 1,372 articles; nine duplicates were removed, 1,363 articles were screened for title and abstract, and 1338 articles were excluded following our pre-defined eligibility criteria. Twenty-five articles underwent full-text screening for possible inclusion in the final qualitative analysis and data extraction; eight articles were included for analysis. We excluded nine articles because they did not discuss racial/ethnic disparity in diagnosis and or care of PAD, one article did not discuss PAD, and seven articles were reviews, case reports, or comments. Full details of the PRISMA flow diagram are shown in Figure 1.

Study Characteristics

Across the included studies, a total of 5,512,632 participants were reported; these data originated from a large independent study that may include overlapping patient populations and comprised 4,784,099 (86.78%) White patients, 576,711 (10.46%) Black patients, 51,408 (0.93%) Hispanic patients, and 100,414 (1.82%) patients from other racial and ethnic groups across the United States. Hispanic and other racial/ethnic minority groups were not mentioned in all the diagnoses and treatment by race/ethnicity in the included articles. The study period ranges from 2009 to 2025. Table 1 shows full details of the study characteristics.

**Table 1:** Study characteristics. USA: United States of America, W: White, B: Black, H: Hispanic, O: other minority tribes.

Author's name	Year	Country	Study design	Sample size
Ferdinand et al. [10]	2023	USA	Cohort	W: 454,382; B: 96,162
Gober et al. [11]	2021	USA	Cohort	W: 17; B: 27
Rowe et al. [12]	2010	USA	Cohort	W: 58,079; B: 17,118; H: 5,590; O: 6,550
O’Donnell et al. [13]	2019	USA	Cohort	W: 74,891; B: 15,572
Amaranto et al. [14]	2009	USA	Cohort	W: 200; O: 53
Meadows et al. [15]	2009	USA	Cohort	W: 1,816; B: 237; H: 115
Secemsky et al. [16]	2025	USA	Cohort	W: 2,037,752; B: 228,257; H: 45,703; O: 93,811
Vogel et al. [17]	2025	USA	Cohort	W: 2,156,962; B: 219,338

Disparity Diagnosis and Management

Only one article discussed diagnostic testing (ankle-brachial index, vascular ultrasound, angiography, and aortography) among different racial groups and reported that Black patients were more likely to undergo diagnostic evaluation for PAD [10]. We identified various treatment modalities for PAD across the included articles, including medical therapy (statins and antiplatelet agents), endovascular/surgical revascularization, vascular bypass surgery, and amputation.

Among the various treatment modalities for PAD, the majority of the White patients underwent revascularization [10,12-14], revascularization and medical therapy [15], vascular bypass surgery [12,13,15], and medical therapy [10,11,15]. In contrast, Black patients more frequently underwent amputation as the treatment of choice for PAD [10,12,13,16,17]. The majority of the included studies used percentages to represent the number of ethnicities who received various interventions for PAD, while Rowe et al. [12] used adjusted odds ratios (AOR) and Secemsky et al. [16] used hazard ratios (HR) as their unit of measurement. Table 2 shows the different management plans individuals in different ethnicities/races underwent; the majority of studies used the percentage, while others used the odds ratio scale.

**Table 2:** Management of PAD with ethnicity/race. W: White, B: Black, H: Hispanic, O: other minority tribes, OR: odds ratio, AOR: adjusted odds ratio, HR: hazard ratio, N/A: not analyzed, PAD: peripheral artery disease.

Author	Intervention	Measure	White (%)	Black (%)	Hispanic (%)	Other (%)	Effect estimation
Ferdinand et al. [10]	Medication only	% + OR	57.0%	13.7%	N/A	N/A	OR: 1.47
Meadows et al. [15]	Medication only	%	77.0%	72.0%	68.0%	N/A	N/A
Ferdinand et al. [10]	Revascularization + medication	% + OR	3.3%	1.0%	N/A	N/A	OR: 1.26
Ferdinand et al. [10]	Revascularization only	% + OR	0.6%	0.1%	N/A	N/A	OR: 0.67
Rowe et al. [12]	Revascularization only	AOR	1.0	0.53	0.66	0.77	AOR > White
O’Donnell et al. [13]	Revascularization only	%	59.0%	54.0%	N/A	N/A	N/A
Amaranto et al. [14]	Revascularization only	%	83.5%	N/A	N/A	16.5%	N/A
Secemsky et al. [16]	Revascularization only	HR	N/A	1.06	N/A	N/A	HR > Black
Rowe et al. [12]	Vascular bypass	AOR	1.0	0.69	0.75	1.02	AOR > White
Meadows et al. [15]	Vascular bypass	%	3.4%	0.6%	5.2%	N/A	N/A
Ferdinand et al. [10]	Amputation	% + OR	48.2%	62.3	N/A	N/A	OR: 1.74
Rowe et al. [12]	Amputation	AOR	1.0	2.49	1.43	1.19	AOR > Black
O’Donnell et al. [13]	Amputation	%	6.0%	16.0%	N/A	N/A	N/A
Secemsky et al. [16]	Amputation	HR	N/A	2.79	N/A	N/A	HR > Black
Vogel et al. [17]	Amputation	%	7.6%	8.9%	N/A	N/A	N/A
Gober et al. [11]	No medication	%	38.0%	62.0%	N/A	N/A	N/A
Ferdinand et al. [10]	No revascularization/medication	%	21.7%	2.7%	N/A	N/A	OR: O.60
Amaranto et al. [14]	No revascularization/medication	%	75.0%	N/A	N/A	25.0%	N/A

Discussion

This systematic review provides an updated synthesis of racial and ethnic disparities in the diagnosis and management of peripheral artery disease (PAD) across the United States from 2009 to 2025. The findings reveal consistent and significant inequities, particularly affecting Black patients, who experience lower rates of revascularization and medical therapy but markedly higher rates of limb amputation compared with White patients. Hispanic and other minority groups remain critically underrepresented in the PAD literature, reflecting a broader pattern of research and clinical neglect.

Racial and Ethnic Disparities in Peripheral Artery Disease (PAD) Diagnosis

Our analysis identified a paucity of data regarding racial and ethnic differences in PAD diagnosis. Only one included study, Ferdinand et al., 2023 [10], explicitly compared diagnostic testing rates, reporting slightly higher use of ankle-brachial index (ABI) and vascular ultrasound among Black patients. However, these differences may not indicate equitable screening, but rather reflect delayed presentation and more advanced disease at diagnosis, a trend repeatedly documented in prior national cohorts [4,7,18]. The limited evidence underscores the need for proactive, community-based screening initiatives, particularly in populations with high cardiometabolic risk burdens.

Disparities in Peripheral Artery Disease (PAD) Management and Outcomes

The most striking pattern across studies was the treatment divergence by race. White patients were significantly more likely to receive evidence-based medical therapy (statins and antiplatelets), endovascular or open revascularization, and limb-salvage procedures. In contrast, Black patients consistently demonstrated higher amputation rates, even after adjustment for comorbidities and socioeconomic status. For instance, Rowe et al. [12] and O’Donnell et al. [13] reported odds ratios exceeding 2.0 for amputation among Black patients, while Secemsky et al. [16] reported a hazard ratio of 2.79 for amputation among Black patients compared with White patients.

These findings echo national data showing that Black Americans are up to four times more likely to undergo major lower-limb amputation than their White patients, even when controlling for diabetes, insurance coverage, and hospital type [4,6,19,20]. The observed disparities in revascularization suggest persistent inequities in referral patterns, provider bias, and access to vascular specialists.

Underrepresentation of Hispanics and Other Minorities

A notable finding from our synthesis was the scarcity of data for Hispanic, Asian, and Native American populations, who collectively comprise a growing proportion of the U.S. population. Only two studies included Hispanics in their analyses, and few stratified treatment outcomes by ethnicity. This omission perpetuates an incomplete understanding of PAD epidemiology and limits the development of equitable interventions. It also highlights an urgent need for improved data collection and racial/ethnic reporting standards in clinical registries and administrative datasets.

Potential Mechanisms and Structural Determinants

The racial disparities observed in PAD management likely arise from a confluence of clinical, socioeconomic, and structural factors. Healthcare access plays a role, as minority patients are more likely to receive care at under-resourced hospitals and less likely to be referred for vascular evaluation before critical limb ischemia develops [20,21]. Provider bias and clinical decision-making are also contributors, as implicit bias may influence the perceived candidacy for revascularization versus amputation [22,23].

Additionally, socioeconomic and insurance disparities, such as lower income, lack of private insurance, and higher prevalence of comorbidities (e.g., diabetes, chronic kidney disease), may reduce opportunities for preventive management and limb salvage [24,25]. Geographic factors, including regional differences in hospital resources and racial composition, also strongly correlate with amputation rates [26,27].

These intersecting determinants align with the framework of structural racism in healthcare, wherein institutional and systemic factors perpetuate unequal treatment access and outcomes [28,29].

Strengths and Limitations

This systematic review possesses several notable strengths that enhance the robustness and interpretability of its findings. First, it represents one of the most comprehensive and contemporary syntheses of racial and ethnic disparities in the diagnosis and management of peripheral artery disease (PAD) across the United States, encompassing studies published between 2009 and 2025. With data drawn from over 5.5 million individuals, the review integrates large-scale administrative and registry-based cohorts that collectively enhance external validity and reflect real-world clinical patterns.

The methodological rigor of the review also stands out. The search strategy adhered strictly to the Preferred Reporting Items for Systematic Reviews and Meta-Analyses (PRISMA 2020) guidelines, ensuring a transparent and replicable process. Moreover, by quantitatively presenting disparities using measures such as odds ratios, adjusted odds ratios, and hazard ratios, the review offers a more precise depiction of the magnitude of inequities compared with prior narrative reviews.

By identifying the underrepresentation of Hispanics, Asians, and Native Americans in PAD literature, the review highlights critical research gaps and sets a clear agenda for future studies that emphasize data disaggregation and inclusive reporting.

Despite these strengths, several limitations must be acknowledged. The included studies were heterogeneous in terms of design, data sources, and outcome measures, limiting comparability and precluding meta-analytic synthesis. Variations in the definitions of PAD, amputation, and revascularization, as well as differences in follow-up duration, may have contributed to measurement inconsistency across studies.

A second limitation concerns racial and ethnic representation. The majority of included studies compared only Black and White populations, with minimal inclusion of other minority groups. Consequently, the review’s conclusions may not fully capture disparities affecting Hispanic, Asian, Native American, or mixed-race populations, groups that often face distinct barriers to vascular care. One of our search databases gave us access to only 50 pages.

Diagnostic disparities were also difficult to assess, as only one study reported racial differences in the use of diagnostic modalities such as the ankle-brachial index or vascular ultrasound. This limited evidence constrains the ability to evaluate disparities at earlier stages of disease detection, where interventions could be most beneficial.

Finally, publication bias cannot be ruled out. Studies reporting significant disparities are more likely to be published than those finding no differences, potentially exaggerating observed effects. Contextual variability, including regional differences in healthcare infrastructure and practice patterns, further complicates the interpretation of national trends.

Recommendations

The findings of this review underscore a pressing need for multi-level interventions to address racial and ethnic disparities in the diagnosis and management of peripheral artery disease (PAD) in the United States. Given the consistent evidence of unequal treatment and disproportionate rates of amputation among Black patients, along with limited data on Hispanic, Asian, and other racial and ethnic groups, efforts should prioritize equity in clinical practice and further investigation of systemic factors associated with these disparities.

At the clinical level, vascular specialists, cardiologists, and primary care providers should incorporate race-conscious, equity-oriented care pathways into PAD management. This includes proactive screening for high-risk populations using the ankle-brachial index, timely referral to vascular specialists, and standardized protocols that ensure all patients receive evidence-based medical therapy, including statins and antiplatelet agents. Hospitals and vascular programs should implement bias-mitigation and equity training to reduce implicit bias in procedural decision-making and referral patterns, particularly regarding revascularization and amputation thresholds.

From a health systems perspective, healthcare institutions should integrate racial and ethnic stratification of quality metrics for PAD care, including revascularization and amputation rates, within performance monitoring frameworks. Transparent reporting and accountability at institutional and regional levels can help identify inequities in care delivery. Expanding access to multidisciplinary limb-preservation clinics in resource-limited and minority-serving hospitals is also critical to reduce preventable amputations.

At the policy level, federal and state health agencies should promote equitable reimbursement structures that incentivize limb-salvage interventions and penalize unwarranted amputations. Investment in preventive vascular screening programs and community-based outreach in high-risk minority neighborhoods can help address delayed diagnosis and reduce disease progression. Funding bodies such as the National Institutes of Health (NIH) should prioritize research that explicitly investigates racial and ethnic differences in PAD care and outcomes, ensuring inclusion of underrepresented groups and intersectional analyses of race, socioeconomic status, and geography.

Finally, at the research and data governance level, future studies should adopt standardized racial and ethnic data collection and reporting across all PAD registries and clinical trials. The limited representation of Hispanic, Asian, and Native American patients in the current literature constitutes a major evidence gap. Longitudinal and mixed-methods designs, integrating patient-reported experiences, access barriers, and provider-level factors are essential to unravel the mechanisms underpinning these disparities and to inform targeted interventions.

## Conclusions

This systematic review highlights persistent and substantial racial and ethnic disparities in the diagnosis and management of peripheral artery disease (PAD) in the United States. According to our study, Black patients consistently experienced higher rates of lower-limb amputation and lower utilization of revascularization and evidence-based medical therapies compared with White patients. Limited data on diagnostic practice and the underrepresentation of Hispanic and other minority populations highlight an important research gap.

## Appendices

Appendix 1 

**Table 3:** Search strategy.

Database	Query	Outcome
PubMed	((((((Ethnic) OR (Racial)) OR (Disparity)) AND (Treatment) AND (Peripheral artery disease)) AND (United States)	372
Google scholar	"Ethnic" "Racial" "Disparity" "Treatment" "Peripheral artery disease" "United States"	1000

Appendix 2

**Table 4:** JBI cohort risk of bias. Y: yes, N: no, JBI: Joanna Briggs Institute.

Checklist question	Ferdinand et al. [10]	Gober et al. [11]	Rowe et al. [12]	O’Donnell et al. [13]	Amaranto et al. [14]	Meadows et al. [15]	Secemsky et al. [16]	Vogel et al. [17]
Were the two groups similar and recruited from the same population?	Y	Y	Y	Y	Y	Y	Y	Y
Were the exposures measured similarly to assign people to both exposed and unexposed groups?	Y	Y	Y	Y	Y	Y	Y	Y
Was the exposure measured in a valid and reliable way?	Y	Y	Y	Y	Y	Y	Y	Y
Were confounding factors identified?	Y	Y	Y	Y	Y	Y	Y	Y
Were strategies to deal with confounding factors stated?	Y	Y	Y	Y	Y	Y	Y	Y
Were the groups/participants free of the outcome at the start of the study (or at the moment of exposure)?	Y	Y	Y	Y	Y	Y	Y	Y
Were the outcomes measured in a valid and reliable way?	Y	Y	Y	Y	Y	Y	Y	Y
Was the follow up time reported and sufficient to be long enough for outcomes to occur?	Y	N	N	Y	N	Y	N	N
Was follow up complete, and if not, were the reasons to loss to follow up described and explored?	Y	N	N	Y	N	Y	N	N
Were strategies to address incomplete follow up utilized?	N	N	N	N	N	N	N	N
Was appropriate statistical analysis used?	Y	Y	Y	Y	Y	Y	Y	Y
